# Effect of stimulant medication on loss of control eating in youth with attention deficit/hyperactivity disorder: a prospective, observational case series study protocol

**DOI:** 10.1186/s40337-022-00674-y

**Published:** 2022-11-01

**Authors:** Aaron R. Keshen, Anja Hilbert, Victoria Taylor, Anastasia L. Harris, Nami Trappenberg, Joseph Sadek, Guido K.W. Frank, Stuart B. Murray

**Affiliations:** 1 Eating Disorder Program, Nova Scotia Health Authority, Halifax, NS Canada; 2 Department of Psychiatry, Dalhousie University, Halifax, NS Canada; 3 Integrated Research and Treatment Center AdiposityDiseases, Behavioral Medicine Research Unit, Department of Psychosomatic Medicine and Psychotherapy, University of Leipzig Medical Center, Leipzig, Germany; 4 Department of Psychiatry, University of California, San Diego, CA USA; 5 Department of Psychiatry and Behavioral Sciences, University of Southern California, Los Angeles, CA USA

**Keywords:** Loss of control eating, Attention deficit/hyperactivity disorder, Stimulants, Eating disorders, Children, Binge eating

## Abstract

**Background:**

Loss of control eating (LOC-E) in youth predicts the later development of full-syndrome binge-eating disorder (BED), and therefore, could be a relevant target for prevention treatments. To develop these treatments, it is important to understand the underlying disease processes and mechanisms. Based on the putative role of neurocognitive impairments in the pathogenesis of LOC-E, treatments that modulate these neurocognitive factors warrant further exploration. For instance, stimulants are an effective treatment for impulsivity in youth with attention deficit/hyperactivity disorder (ADHD) and have been shown to improve symptoms of BED in adults. Notably, stimulants have not been examined as a treatment for LOC-E in youth. To explore this gap, we aim to measure change in LOC-E episodes and secondary outcomes in youth with comorbid ADHD and LOC-E who are being started on stimulants.

**Methods:**

We will collect prospective observational data on forty 8-to-13-year-old youth diagnosed with comorbid ADHD and LOC-E who are initiating a stimulant for ADHD. Prior to stimulant initiation, participants will complete baseline measures including LOC-E episode frequency in the last 3 months (primary outcome), and secondary outcomes including disordered eating cognitions, emotions and behaviors, ADHD symptom severity, parental LOC-E, impulsivity and reward sensitivity, and anxiety/mood severity. Outcome measurements will be gathered again at 3-months after initiating the stimulant. Within-patient standardized effect sizes with 95% confidence intervals will be calculated from baseline to 3-month follow-up for all outcomes.

**Discussion:**

Many individuals with LOC-E or binge eating do not fully remit over the course of psychotherapy. Whereas psychotherapy may address psychological and sociocultural domains associated with LOC-E, some individuals with neurocognitive impairments (e.g., ADHD) and neurobiological deficits (e.g., low intrasynaptic dopamine or norepinephrine) may benefit from adjunctive treatment that targets those factors. This will be the first study to provide pilot data for future studies that could examine both the effect of stimulants on LOC-E in youth and underlying mechanisms.

**Trial registration:**

Trial registration number: NCT05592119

## Background

Binge-eating episodes are defined as the consumption of an objectively large amount of food while experiencing a loss of control over eating [1]. In children, accurately quantifying a “large amount of food” is problematic because intermittent overconsumption is a normal behavior in growing youth [2]. Instead, many researchers measure ‘loss of control eating’ (LOC-E) rather than binge eating when studying disordered eating in youth. Notably, 9–30% of 9–13-year-old youth experience LOC-E, though only 1/3 to 1/2 of these children present with these symptoms after 5 years [3]. In addition to a high prevalence rate, LOC-E in middle childhood (8–12 years old) also robustly predicts the later development of partial or full-syndrome binge-eating disorder (BED) [4–6] and excessive weight gain in children at high risk for adult obesity [4, 7]. Based on these factors, LOC-E has been proposed as a relevant target for preventive interventions [8].

To develop treatments for LOC-E, it is important to understand the underlying disease processes and mechanisms. Recent evidence suggests that neurocognitive predisposing factors (e.g., impulsivity and dysfunctional reward processing) are relevant in the pathogenesis of LOC-E. First, there is evidence that children (8–14 years old) with Attention Deficit/Hyperactivity Disorder (ADHD) have 12 times the risk of experiencing LOC-E compared to healthy controls [9]. This study also found that children with LOC-E are more likely to exhibit high impulsivity on a neurobehavioral task (*Go/No Go*) and parent-rated scale compared to healthy controls. Another study used a delay of gratification task (DoGT) in children with ADHD or LOC-E, comorbid diagnoses (ADHD and LOC-E), and healthy controls [10]. They found that children with ADHD and comorbid diagnoses had significantly higher risk of eating prematurely during the DoGT compared to children with only LOC-E or healthy controls. Moreover, the children with comorbid diagnoses were most likely to worry about losing control over eating during the task. These findings suggest that a lack of behavioral inhibition (to food reward) in children with ADHD is a relevant factor in the drive to eat prematurely [10].

Neurobiological studies have shown that the relationship between ADHD and binge eating (in adults) is largely explained by shared genetic risk factors [11]; however, there are also non-shared environmental factors that explain the covariance [8]. In a neuroimaging study, Murray et al. [12] found that children with early onset BED have underlying dysconnectivity between the brain’s inhibitory network and reward system. These findings suggest that poor connectivity between reward and impulse-control-related neural pathways underlie some children’s inability to regulate their drive to eat. Importantly, similar perturbations in functional connectivity have been illustrated in key nodes of the inhibitory control network in those with ADHD [13] – suggesting similar underlying mechanisms.

Recent research has started to delineate not only the association between ADHD and LOC-E, but also the directionality of the relationship. A large longitudinal study by Sonneville et al. [14] showed that ADHD in children (mean = 11.7 years old) predicted binge eating in mid-adolescence (14–16 years old) via late childhood overeating and early adolescent ‘strong desire for food’. Relatedly, emerging longitudinal data are supporting a causal role of ADHD in contributing to excessive weight gain [15].

Considering this evidence, it is relevant to examine whether modifying neurocognitive symptoms, such as impulsivity, influences LOC-E and the subsequent development of eating disorders and obesity. Stimulant medication is an example of a treatment that could modify these neurocognitive symptoms, and in doing so, potentially effect change in symptoms of LOC-E. Robust evidence supports stimulants as an efficacious treatment for impulsivity in youth with ADHD [16]; however, studies have not explored the effect of stimulants on LOC-E in children. Relevantly though, there is evidence that stimulants improve symptoms of binge eating in adults, and also, that stimulants may mediate this effect by improving impulsivity [17–19]. Notably, the stimulant lisdexamfetamine (LDX) received regulatory approval for the treatment of moderate to severe BED in adults based on one 11-week Phase 2 dose-finding study and two identically designed 12-week Phase 3 dose optimization studies [19–21]. Across the Phase 3 trials, LDX (50 and 70 mg/day) was superior to placebo in decreasing binge eating days/week and improving key secondary outcomes (e.g., binge cessation) over 12-weeks [19].

In addition to clinical trial evidence, there are also neurobiological studies that support the use of stimulants as a treatment for LOC-E [22, 23]. For example, explanatory models for ADHD suggest that reduced dopamine neuronal tone affects executive functioning and reward processing [24]. Similarly, disordered eating, such as binge eating has been associated with lower dopamine (and norepinephrine) metabolite levels in the cerebrospinal fluid [25, 26]. In sum, for ADHD and eating disorders, there is evidence of altered dopaminergic tone, presumably *lower* intrasynaptic dopamine, and since stimulant medications *increase* intrasynaptic dopamine and norepinephrine levels in striatal and cortical brain regions, a plausible explanatory model supports the use of stimulant medication for LOC-E.

As further support for exploring the effect of stimulants on LOC-E in youth, retrospective and cross-sectional data suggests that children with ADHD who had been treated with stimulants had less odds of obesity compared to those who were not medicated [27]. Importantly, the research does not elucidate whether stimulants were associated with lower obesity rates because they lessened symptoms of LOC-E, or alternatively, whether a different mechanism mediated the apparent effect on obesity (e.g., appetite suppression independent of effect on disordered eating).

In sum, though stimulants warrant investigation as a treatment for LOC-E in youth, no studies have prospectively examined the effect of stimulants on this form of disordered eating. To gather pilot data, we aim to measure prospective, observational outcomes in a clinical setting to examine the effect of stimulant initiation on LOC-E in children with comorbid ADHD and LOC-E. We will also collect exploratory secondary outcomes to measure eating-related cognitions, emotions and behaviors, ADHD symptom severity, parental LOC-E, impulsivity and reward sensitivity, and anxiety/mood symptoms. Given the observational design, we will not generate a priori hypotheses.

## Methods

### Design

This study will use a prospective, observational case series design. Ethics approval was obtained through the Nova Scotia Health Research Ethics Board.

### Participants

We will collect data on forty 8–13-year-old youth and their parents. The study will use a prospective cohort sample from the Atlantic ADHD Centre in Dartmouth, Nova Scotia. To be eligible for inclusion, youth must (A) meet the diagnostic criteria for ADHD, and (B) have experienced at least 3 episodes of LOC-E during the past 3 months^1^, accompanied by some degree of distress and 2 of the 5 behavioral symptoms associated with LOC-E. Inherent to the definition of LOC-E, consumption of an abnormally large amount of food will not be required. Further details related to the diagnostic assessment for ADHD and LOC-E are provided in later sections. Participants will be excluded if they are (a) currently receiving psychotherapy treatments for ADHD, eating disorders, or obesity, and/or (b) undergoing concurrent medication changes that could affect ADHD or eating related symptoms (as determined by psychiatrist and principal investigator [Aaron Keshen]). Participants must also have sufficient language skills and a general practitioner to initiate the stimulant and monitor adverse events.

### Measures

**Fig. 1:**
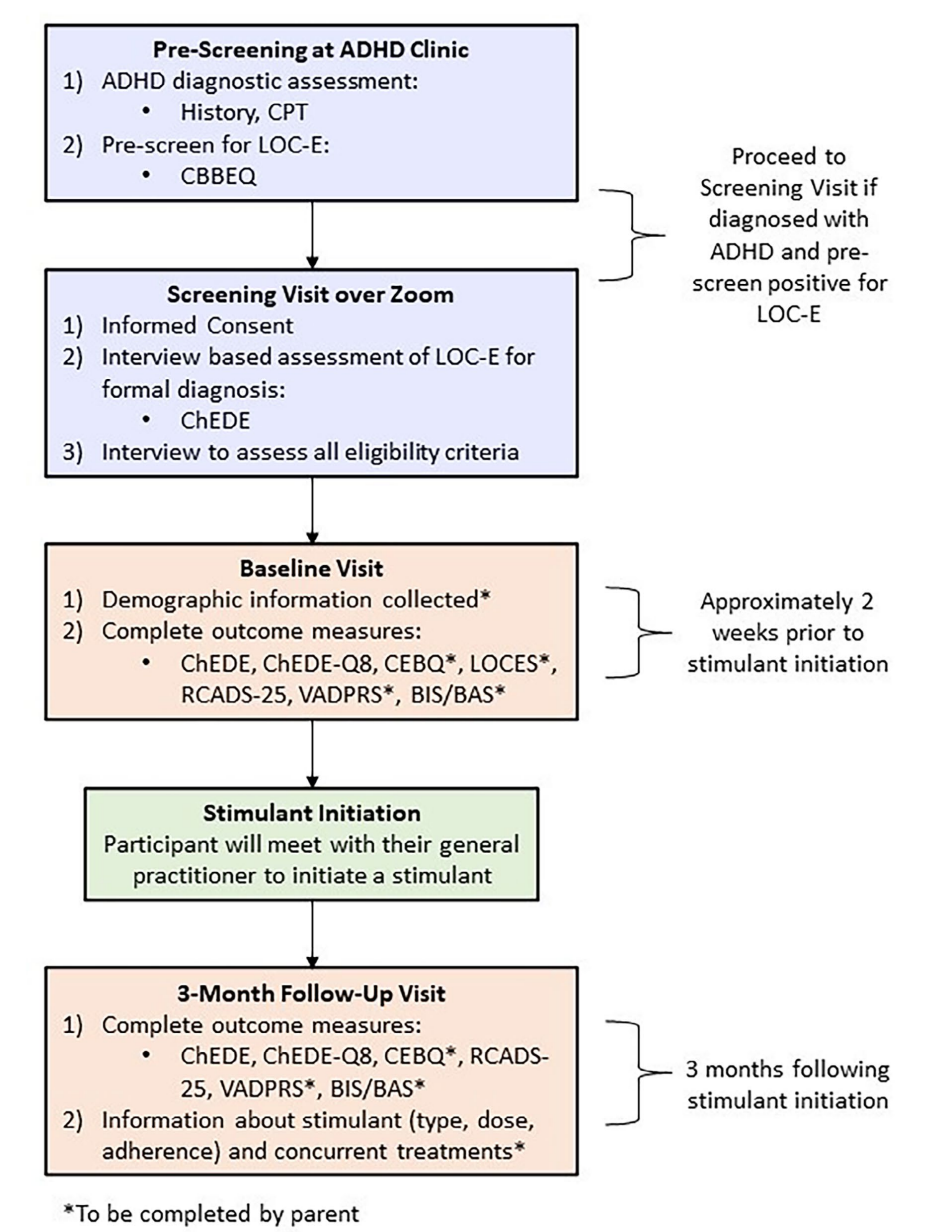
Timeline of Study Procedures. *CPT* Continuous Performance Test. *CBBEQ* Children’s Brief Binge-Eating Questionnaire. *ChEDE* Eating Disorder Examination – Child Version. *ChEDE-Q8* Eating Disorder Examination Questionnaire adapted for children, short form. *CEBQ* Child Eating Behavior Questionnaire. *LOCES* Loss of Control Over Eating Scale. *RCADS-25* Revised Child Anxiety and Depression Scale – Short Version. *VADPRS* Vanderblit ADHD Diagnostic Parent Rating Scale. *BIS/BAS* Parent-Report Behavioral Inhibition System/Behavioral Activation System Scales

The following outcome measures will be collected at various time points including the pre-screening visit, screening visit, baseline, and 3-month follow-up. Figure 1 illustrates an overview of these study visits.

#### Children’s brief binge-eating Questionnaire (CBBEQ)

The CBBEQ is a 7-item screening tool for binge-eating symptomatology that has been validated for use with children aged 7 and above [29]. In this self-report measure, level of agreement with statements concerning eating behaviors and thoughts regarding food are rated on a 4-point Likert scale from 0 (definitely false) to 3 (definitely true). The global mean score of the CBBEQ will be used, with higher scores indicating greater levels of global binge-eating symptomatology. The CBBEQ has high internal consistency and concurrent validity, and scores above 8 warrant a more extensive interview for diagnostic assessment [29].

#### Vanderbilt ADHD diagnostic parent rating scale (VADPRS)

The VADPRS [30], which is to be completed by the parent of the child under assessment, is a 56-item measure used to evaluate ADHD behaviors, along with academic and social performance in children aged 6 and above. The VADPRS incorporates all DSM-5 criteria for both ADHD subtypes [31], and is divided into two components: one which specifically assesses ADHD symptoms, and the other examines impairment in performance. The ADHD symptoms component consists of two ADHD subscales, inattention and hyperactivity/impulsivity, as well as three subscales for common comorbidities, including oppositional defiant disorder, conduct disorder, and anxiety/depression. Scores range from 0 (never) to 3 (very often) on a 4-point Likert scale, with higher scores indicating greater levels of symptoms exhibited by the child. The performance component has scores ranging from 1 (excellent) to 5 (problematic) on a 5-point scale, with higher scores indicating greater impairment in academic and/or social performance. The total scores from each subscale of the symptomatology component will be used in combination with the score of the impairment in performance component. The VADPRS has demonstrated good construct, discriminant, and content validity, as well as internal consistency [30, 32–34].

#### Eating disorder examination – child Version 17.D/C.1 (ChEDE) – diagnostic item 11

The EDE 17.0D [35], which measures eating disorder symptom severity, has been adapted for use with children ages 8 and above [36, 37]. The ChEDE has been shown to have high inter-rater reliability and internal consistency [38, 39]. For the purposes of this study, only diagnostic item 11 (identifying bulimic episodes and other episodes of overeating) of the ChEDE will be used, which includes questions regarding feelings of loss of control over eating, features associated with binge eating, and distress about binge eating. The measure will be used for diagnostic assessment of LOC-E for study inclusion purposes, and at a baseline and 3-month follow-up to establish LOC-E episode frequency. The ChEDE will be administered by well trained research assistants tasked to develop positive rapport with the children and ensure concepts are explained in a manner that is developmentally appropriate to each child [see 40 for a detailed description]. Also, parents will remain present during the assessment in case the children need assistance understanding the interviewer.

#### Short form eating disorder examination questionnaire adapted for children (ChEDE-Q8)

The Child Eating Disorder Examination-Questionnaire (ChEDE-Q) is a self-report version of the Child Eating Disorder Examination (ChEDE). The English version of the ChEDE-Q8 has been derived from the German version of the ChEDE-Q8 [40] and the English version of the ChEDE-Q [41]. The ChEDE-Q8 is an 8-item self-report measure that evaluates four areas of eating disorder psychopathology: restraint, eating concern, shape concern, and weight concern. ChEDE-Q8 scores range from 0 (absence of the feature) to 6 (feature present every day or to an extreme degree). Global mean scores will be used, with higher scores indicating higher levels of eating disorder psychopathology. The ChEDE-Q8 was validated for children ages 7 and above and has been shown to have strict measurement invariance, internal consistency, and convergent as well as factorial validity [40].

#### Child eating Behavior Questionnaire (CEBQ)

The CEBQ [42], which is to be completed by the parent of a child under assessment, is a 35-item measure used to evaluate cognitions, emotions, and behaviors associated with disordered eating. Eight aspects of eating behavior are assessed: food responsiveness, emotional over- and undereating, enjoyment of food, desire to drink, satiety responsiveness, slowness in eating, and food fussiness. The CEBQ is rated on a 5-point Likert scale with scores ranging from 1 (never) to 5 (always). The eight subscale scores and global mean score will be used for analysis, with higher scores indicating a greater level of disordered eating. The CEBQ has been shown to have high test-retest reliability and is a valid method of assessing obesogenic eating behaviors [42, 43].

#### Loss of Control Over Eating Scale (LOCES)

The LOCES [44], which is to be completed by parents in relation to their own eating psychopathology, is a 24-item self-report measure used to examine three aspects of LOC-E: behavioral, cognitive/dissociative, and positive/euphoric. The LOCES consists of three subscales based on the three aspects of LOC-E, with scores ranging from 1 (never) to 5 (always) on a 5-point Likert scale. The global mean score calculated from the average of each subscale will be used, with higher scores indicating greater levels of LOC-E. The LOCES has been shown to have high internal consistency, convergent and discriminant validity, and test-retest reliability [44].

#### Parent-report behavioral inhibition System/Behavioral activation system scales (BIS/BAS)

The BIS/BAS [45] is a 20-item parent-report measure (examining their child’s behavior), adapted from the original self-report BIS/BAS scales [46], which examines two motivational systems through responses to reward and punishment. The BIS/BAS contains four subscales: the inhibition system (BIS), reward responsiveness (BAS), drive (BAS), and fun seeking (BAS). Impulsivity has been found to be positively correlated with BAS-drive and BAS-fun seeking, and negatively correlated with BIS [47], therefore, the BIS/BAS can be used as a proxy for impulsivity in youth. Seven questions related to sensitivity to punishment are used to assess the BIS, and thirteen questions related to sensitivity to reward to assess the BAS; scores range from 1 (not true at all) to 4 (all true) on a 4-point Likert scale. The total separate scores of the four subscales will be used, with higher scores indicating greater levels of sensitivity in the associated motivational system. The parent-report BIS-BAS is a reliable and valid tool for examining punishment and reward sensitivity in children [45].

#### Revised child anxiety and Depression Scale – Short Version (RCADS-25)

The RCADS-25 [48] is a 25-item self-report measure, adapted from the original 47-item RCADS [49, 50], used to assess depression and anxiety symptoms in children. The RCADS-25 contains 10 questions based on major depressive disorder, and 15 questions based on five DSM-IV anxiety domains: separation anxiety disorder, generalized anxiety disorder, panic disorder, social phobia, and obsessive-compulsive disorder. RCADS-25 scores range from 0 (never) to 3 (always) on a 4-point Likert scale. Global anxiety and depression scores will be used separately, with higher scores on each scale indicating greater levels of anxiety and depression. The RCADS-25 is a reliable clinical tool and has been shown to have discriminant, convergent, and divergent validity [51].

### Primary outcome

LOC-E episode frequency in the last 3-months is the primary outcome of this study, as measured by the ChEDE.

### Secondary outcomes

The secondary outcomes of this study include disordered eating cognitions, emotions, and behaviors (measured using the ChEDE-Q8 and CEBQ), ADHD symptom severity (measured using the VADPRS), impulsivity and reward sensitivity (measured using the BIS/BAS), and anxiety/mood severity (measured using the RCADS-25).

### Procedure

Clinicians at the ADHD clinic will routinely pre-screen for LOC-E, using the CBBEQ [29], as part of their standard clinical assessment (see Fig. 1 for schedule of assessments). The clinic’s standard ADHD diagnostic assessment includes a psychiatric history by an ADHD expert (JS) and cognitive data using a validated Continuous Performance Test (CPT). Patients who are diagnosed with ADHD and pre-screen positive for LOC-E (score of 8 or above on the CBBEQ), and who receive a recommendation to start a stimulant medication and intend to initiate the stimulant, will be invited to the consent and screening visit. Pre-screening data collection is standard practice at the clinic (i.e., relevant personal health information and scale-based outcomes) and will not be recorded for the study as consent will not have been obtained. However, clinic staff will record the following non-personal health information that can be used in a participant flow diagram for study purposes: (a) number of positive and negative pre-screens, and (b) number of patients who are interested or not interested in being contacted by a research staff to learn more about the study.

Parents and children who are interested in participating will meet with a research assistant over Zoom for Healthcare to review the consent form and undergo a more detailed screening visit to establish eligibility. During the screening visit a trained research assistant will use the diagnostic version of the ChEDE to confirm the LOC-E inclusion criteria are met. If the LOC-E diagnosis is confirmed and no exclusion criteria are met, the child’s parent will be instructed to contact the research assistant approximately 2 weeks prior to stimulant initiation (which will occur naturalistically in the child’s physician’s office outside of the study context). At that time, participants will meet with a research assistant again to obtain baseline measurements (i.e., prior to stimulant initiation) and demographic information (age, sex, previous and current psychological and pharmacological treatments, and parent reported comorbid diagnoses of physical and psychiatric conditions). Outcome measurements will be gathered again at 3-months after initiating the stimulant, as will information about the stimulant treatment (i.e., type, dose, adherence, and adverse events [adapted from the Pittsburgh Side-Effects Rating Scale]) [52]. Participants will be paid $25 per visit after the final study visit.

### Data analytic plan

Within-patient standardized effect size information (i.e., Cohen’s d and 95% confidence intervals) from baseline to 3-month follow-up will be calculated for LOC-E episodes and all secondary outcomes. Clinical outcome data will be analysed for all youth who remain on a stimulant for the 3-month duration. Descriptive statistics (e.g., means and standard deviations) will be used to report demographic information, medication doses, adverse events, and frequency of medication discontinuation. If available, reasons for medication discontinuation and loss to follow-up will be documented. Given the case series design, there will be no a priori power analysis.

## Discussion

This will be the first study to prospectively examine the effect of stimulants on LOC-E in youth. The limitations of this observational design include the inability to control for placebo or time effects and other confounding variables (e.g., variation in medication type and dose). Other limitations include the small sample size, short treatment duration, and risk of self-report, assessment, and recall biases (especially given the age of our sample).

Those limitations notwithstanding, this study explores a novel intervention that could lead to important future research. If our results show that stimulants are associated with a decrease in LOC-E over 3-months, the effect size data could be used for a power calculation in future clinical trials. A hypothetical future direction could include a two-phase randomized controlled trial to compare a group of youth with comorbid ADHD and LOC-E treated with a psychotherapeutic intervention to a group treated with a stimulant only. If the psychotherapy group continues to experience LOC-E after the initial treatment phase, the unremitted patients could be crossed over into a stimulant phase to examine whether stimulants benefit individuals who do not respond to psychotherapy.

Investigating stimulants as a possible adjunct for psychotherapy is relevant because only 40–50% of adults treated for LOC-E (or binge eating) remit with psychological treatments alone [53]. In one of the few psychotherapy trials for pre-adolescent children with LOC-E, about 40% continued to experience LOC-E after a family-based therapy [54]. One explanation for inadequate response to psychotherapy is that LOC-E is caused by multiple factors, which vary amongst individuals; and therefore, current psychotherapies may not target *all* the putative causal mechanisms. Byrne et al. [8] describe a domain summary of factors related to pediatric LOC-E that includes: (a) genetic/physiological (e.g., hormonal, family history of obesity, neural functioning), (b) socioenvironmental (e.g., dieting, media thin-ideal, peer/family pressure to be thin), (c) psychological (e.g., negative affect, emotion dysregulation, attentional biases, executive function impairment), and (d) behavioral (e.g., impulsivity, intake of energy-dense foods). Within these domains, stimulant medication as an adjunctive treatment may be effective in relation to mechanisms such as neural functioning, executive function impairment, and impulsivity—especially in youth with impairments in these areas (e.g., those with ADHD). Relatedly, another strength of this study is the preliminary exploration of several of these mechanisms. Specifically, the secondary outcomes related to impulsivity, eating-related cognitions, emotions and behaviors, and mood/anxiety will provide pilot data for future studies that examine not only treatment effect, but also underlying mechanisms associated with LOC-E.

## Data Availability

Not applicable.

## Footnotes

1 Three months was chosen because LOC-E episodes in children tend to occur less frequently compared to adults so an average over three months at baseline and follow-up time points will allow for a more meaningful comparison. This approach has been used previously [28].

## List of abbreviations

ADHD: Attention deficit/hyperactivity disorder
BED: Binge-eating disorder
BIS/BAS: Behavioral inhibition system/behavioral activation system scales
CBBEQ: Children’s brief binge-eating questionnaire
CEBQ: Child eating behavior questionnaire
ChEDE: Eating disorder examination – child version C.1
ChEDE-Q8: Eating disorder examination-questionnaire adapted for children, short form
LOC-E: Loss of control eating
RCADS-25: Revised child anxiety and depression scale – short version
VADPRS: Vanderbilt ADHD diagnostic parent rating scale
